# Insight into the resilience and susceptibility of marine bacteria to T6SS attack by *Vibrio cholerae* and *Vibrio coralliilyticus*

**DOI:** 10.1371/journal.pone.0227864

**Published:** 2020-01-28

**Authors:** Ryan Guillemette, Blake Ushijima, Mihika Jalan, Claudia C. Häse, Farooq Azam

**Affiliations:** 1 Marine Biology Research Division, Scripps Institution of Oceanography, University of California San Diego, La Jolla, San Diego, CA, United States of America; 2 Carlson College of Veterinary Medicine, Oregon State University, Corvallis, OR, United States of America; National Institute of Technology Rourkela, INDIA

## Abstract

The type VI secretion system (T6SS) is a nanomachine capable of killing adjacent microbial cells in a contact-dependent manner. Due to limited studies, relatively little is known about the range of marine bacteria that are susceptible to T6SS attack. Here, 15 diverse marine bacterial isolates from the phyla Bacteroidetes and Ɣ-Proteobacteria were challenged against the marine bacterium and human pathogen, *Vibrio cholerae*, which has a well described T6SS. *V*. *cholerae* killed several of the tested Ɣ-Proteobacteria, including members of the orders Vibrionales, Alteromonadales, Oceanospirillales, and Pseudomonadales. In contrast, *V*. *cholerae* co-existed with multiple Bacteroidetes and Ɣ-Proteobacteria isolates, but was killed by *Vibrio coralliilyticus*. Follow-up experiments revealed that five *V*. *coralliilyticus* strains, including known coral and shellfish pathogens survived the T6SS challenge and killed *V*. *cholerae*. By using predicted protein comparisons and mutagenesis, we conclude that *V*. *coralliilyticus* protected itself in the challenge by using its own T6SS to kill *V*. *cholerae*. This study provides valuable insight into the resilience and susceptibility of marine bacteria to the *V*. *cholerae* T6SS, and provides the first evidence for a functional T6SS in *V*. *coralliilyticus*, both of which have implications for human and ocean health.

## Introduction

Bacterial-bacterial antagonism plays a major role in shaping bacterial community structure and function [1–5]. Early studies investigating marine bacterial-bacterial antagonism predominantly focused on the production and release of antibiotics by predatory bacteria as a means to inhibit their preys’ growth [6–8]. While these findings demonstrated that select marine bacteria were capable of killing other bacteria, it has been suggested that the relatively low frequency of killing that was observed may have been due to the common use of non-marine bacteria as model prey [9]. Later, experiments that used more ecologically relevant model prey (e.g. isolates from pelagic seawater, marine particles, and coral) found that killing occurred in > 50% of the competition assays [9, 10]. Interestingly, these studies also showed that some of the model prey were able to survive the challenge against select predatory bacteria that had killed other bacteria, suggesting that those surviving prey possessed defense mechanisms [9, 10].

In addition to chemical-mediated bacterial antagonism, marine bacteria possess and use a variety of contact-dependent killing mechanisms [11–13]. In this work, we focused on one such mechanism that is well-characterized and carried by many gram-negative bacteria, the type VI secretion system (T6SS) [12, 14]. The T6SS is a nanomachine that is capable of killing eukaryotic and bacterial prey by directly injecting toxic effector proteins into them, which then carry out a variety of lethal functions [15–18]. Generally, the needle-like apparatus is assembled in stages, and once complete, it resembles an inverted bacteriophage tailspike [19]. Upon assembly initiation, a transmembrane baseplate is formed to anchor the system to the cell envelope [20]. VgrG and PAAR-domain-containing effector proteins are then recruited to the baseplate to form a needle-like tip and serve as the nucleation site for the formation of an Hcp protein tube [21–23]. A sheath comprised of VipA and VipB subunits then assembles around the tube and when the sheath contracts the Hcp/VgrG/PAAR complex is propelled into adjacent target cells [24–27]. Assisting with the extracellular secretion of these effector molecules are the proteins VasK and VasF, which are believed to be associated with the membrane-associated complexes [28]. The ATPase ClpV then disassembles the sheath, and possibly the entire apparatus, within seconds after “firing” the T6SS [29]. Bacteria carrying a functional T6SS exhibit a remarkable ability to efficiently kill their bacterial prey, which can lead to the displacement of host associated commensals [30], intraspecific competition during host colonization [31], community phase separation [32], and possibly intraguild predation [33]. Conversely, some bacteria have developed mechanisms to resist T6SS attack. For example, a recent study demonstrated that the production of exopolysaccharide (EPS) by *Vibrio cholerae* can act as a unidirectional barrier to protect itself from T6SS-mediated predators [34]. Furthermore, it has been documented that some bacteria possess immunity genes against various effectors, which is also how bacterial predators protect themselves against their own T6SS effectors [35–37]. In addition to passive resistance mechanisms, bacteria such as *P*. *aeruginosa* can sense exogenous T6SS attacks and retaliate with a T6SS of their own [38].

Despite the growing number of T6SS studies, relatively little is known about the effectiveness of T6SS deployment against different marine bacteria. It was found that the marine bacterium and human pathogen, *Vibrio cholerae*, is capable of using its T6SS to kill species such as *V*. *communis*, *V*. *harveyi*, *Pseudoalteromonas phenolica*, and *Aeromonas sp*. [39, 40]. Other *Vibrio* species, such as *V*. *parahaemolyticus*, *V*. *alginolyticus*, and *V*. *fischeri* were also shown to have functional T6SSs, however the known scope of their marine prey is restricted to three *Vibrio* species (*V*. *cholerae*, *V*. *natriegens*, and select strains of *V*. *fischeri*) due to a limited number of studies [31, 41, 42]. We considered that further exploration into the range of marine bacteria that are susceptible to the T6SS should increase our understanding of the types of bacteria that a specific T6SS can kill, while also helping to inform microbial ecologists on select types of bacteria, and ultimately the mechanisms, that provide resistance to T6SS attack. Such knowledge may prove useful in understanding marine microbial community dynamics and has already been posited to be an important consideration for the development of antimicrobials and probiotics [34]. Here, we challenged a number of phylogenetically diverse marine bacterial types, including members of the phyla Proteobacteria and Bacteroidetes against T6SS attack from *V*. *cholerae* strain 2740–80. The presented results are broadly discussed in the context of marine microbial ecology, which includes implications for human health, aquaculture, and coral disease research.

## Materials & methods

### Bacterial strains

The phylogeny, description, and source of each isolate that was used in the challenge assays are contained in Tables 1 and 2. Prior to our experiments, each of the marine bacterial challengers listed in Table 2 was plated onto rifampicin containing media to generate spontaneous rifampicin mutants (R^r^). Single R^r^ colonies for each isolate were picked, streaked purified, and confirmed resistant to rifampicin before storage in 25% glycerol at -80°C. For challenge assays, the isolates were grown with Zobell 2216E at 25°C. Autoclaved Zobell 2216E media was prepared by amending 0.22 μm-filtered seawater with 5g of peptone and 1g of yeast extract liter^-1^, while plates contained an additional 15g of agar liter^-1^[43]. The following concentrations of antibiotics were used where appropriate: streptomycin, 100 μg/ml; rifampicin, 50 μg/ml; ampicillin 100 μg/ml (Sigma-Aldrich; St. Louis, MO, USA).

**Table 1:** *V*. *cholerae* and *V*. *coralliilyticus* strains used in this study.

Genus, species, strain		Description	Source/citation
**Wild type**	*V*. *cholerae* 2740–80	Nontoxinogenic El Tor strain isolated from a patient in Florida, United States; Sm^R^, Rf^R^	(Goldberg & Murphy 1983)[44]
	*V*. *coralliilyticus* ATCC BAA-450	Type strain of *V*. *coralliilyticus*; coral pathogen isolated off the coast of Zanzibar; Ap^R^	(Ben-Haim & Rosenberg 2002)[45]
	*V*. *coralliilyticus* OCN008	Coral pathogen isolated from Kaneohe Bay, HI; Ap^R^	(Ushijima et al. 2014)[46]
	*V*. *coralliilyticus* OCN014	Coral pathogen isolated from Palmyra Atoll; Ap^R^, Sm^R^	(Ushijima et al. 2016)[47]
	*V*. *coralliilyticus* RE22	Oyster larvae pathogen isolated from Netarts Bay, OR; Ap^R^, Sm^R^	(Estes et al. 2004)[48]
	*V*. *coralliilyticus* RE98	Oyster larvae pathogen isolated from Netarts Bay, OR; Ap^R^	(Estes et al. 2004)[48]
**Mutant**	*V*. *cholerae* 2740–80 Δ*vipA*	*V*. *cholerae* 2740–80 with an in-frame deletion of *vipA*; T6SS^-^ mutant, Sm^R^, Rf^R^	(Basler et al. 2012)[29]
	*V*. *coralliilyticus* OCN008 Δ*vtpR*	OCN008 with an in-frame deletion of the quorum sensing regulatory protein-encoding gene *vtpR*; Ap^R^	This study
	*V*. *coralliilyticus* OCN008 Δ*vtpA*	OCN008 with an in-frame deletion of the metalloprotease-encoding gene *vtpA*; Ap^R^	This study
	*V*. *coralliilyticus* OCN008 Δ*vtpB*	OCN008 with an in-frame deletion of the metalloprotease-encoding gene *vtpB*; Ap^R^	This study
	*V*. *coralliilyticus* OCN008 Δ*vtpAB*	An OCN008 double deletion mutant with in-frame deletions of *vtpA* and *vtpB*; Ap^R^	This study
	*V*. *coralliilyticus* OCN008 Δ*vasK*	OCN008 with an in-frame deletion of a *vasK* homolog predicted to encode a T6SS-associated protein; Ap^R^	This study

*Abbreviations: Ap^R^ = resistant to ampicillin, Sm^R^ = resistant to streptomycin, Rf^R^ = resistant to rifampicin, Km^R^ = resistant to kanamycin.

**Table 2:** Marine bacterial strains used as challengers in this study.

Phylum, class	Order	Genus, species, strain	Description	Source/citation
Ɣ-Proteobacteia	Vibrionales	*V*. *coralliilyticus* OCN008	Coral pathogen isolated from Kaneohe Bay, HI; Ap^R^	(Ushijima et al. 2014)[46]
		*Vibrio shilonii* AK1	Coral pathogen; Rf^R^	(Kushmaro et al. 1996)[49]
		*Vibrio harveyi* B392	Free-living and marine organism associated bacterium; Rf^R^	(Byers & Meighen 1985)[50]
		*Vibrio* sp. SWAT3	Particle-attached bacterium, isolated from Scripps Pier, CA; Rf^R^	(Long & Azam 2001)[9]
	Alteromonadales	*Pseudoalteromonas* sp. Tw7	Particle-attached bacterium, isolated from Scripps Pier, CA; Rf^R^	(Bidle & Azam 2001)[51]
		*Pseudoalteromonas* sp. Tw2	Particle-attached bacterium, isolated from Scripps Pier, CA; Rf^R^	(Bidle & Azam 2001)[51]
		*Alteromonas* Alt-SIO	Free-living bacterium, isolated from Scripps Pier, CA; Rf^R^	(Pedler et al. 2014)[52]
		*Pseudoalteromonas flavipulchra* 2ta6	Coral associated bacterium that exhibits high antagonism towards other bacteria; Rf^R^	(Rypien et al. 2010)[10]
	Oceanospirillales	*Halomonoas* sp. 73	Isolated from Mariana Trench benthic boundary water; Rf^R^	(Peoples et al. 2018)[53]
	Pseudomonadales	*Pseudomonoas* sp. 28	Isolated from Mariana Trench sediment; Rf^R^	(Peoples et al. 2018)[53]
		*Psychrobacter aquimaris*	Isolated from the South Sea in Korea; Rf^R^	(Yoon et al. 2005)[54]
Bacteroidetes	Flavobacteriales	*Flavobacteria* sp. BBFL7	Isolated from Scripps Pier, CA; Rf^R^	(Bidle & Azam 2001)[51]
		*Salgentibacter* sp. 1	Mariana trench water column; Rf^R^	(Peoples et al. 2018)[53]
		*Aequorivita* sp. 97	Mariana trench sediment; Rf^R^	(Peoples et al. 2018)[53]
	Flammeovirgacea	*Roseivirga* sp. 121	Mariana trench sediment; Rf^R^	(Peoples et al. 2018)[53]

*Abbreviations: Rf^R^ = resistant to rifampicin.

For mutagenesis, *V*. *coralliilyticus* strains were grown in a modified glycerol artificial seawater (GASW) media supplemented with 50 mM Tris-Base (Sigma-Aldrich) (GASW-Tris) and the pH adjusted to 8.3 with HCl prior to autoclaving to prevent acidification of the media and incubated at 27°C[55], unless otherwise stated. For solid media, 15 g/l of agar (Teknova; Hollister, CA, USA) was added prior to autoclaving. All *E*. *coli* strains were grown in LB-Miller at 37°C, unless otherwise stated. Antibiotics for selection with *E*. *coli* were used at the following concentrations unless otherwise stated: kanamycin, 50 μg/ml; streptomycin, 25 μg/ml; spectinomycin, 50 μg/ml; and chloramphenicol, 15 μg/ml (Sigma-Aldrich). Antibiotics for selection with *V*. *coralliilyticus* were used at the following concentrations unless otherwise stated: ampicillin, 200 μg/ml; streptomycin, 50 μg/ml; spectinomycin, 100 μg/ml; and chloramphenicol, 5 μg/ml (Sigma-Aldrich). Growth media for *E*. *coli* auxotrophic strains were supplemented with deoxythymidine (DT) or diaminopimelate (DAP) at a final concentration of 0.3 mM as required (Sigma-Aldrich). Arabinose-induced expression of the *ccdB* gene was achieved by the addition of 0.3% L-arabinose to GASW-Tris (GASW-ARA) and expression was repressed by the addition of 1% D-glucose to LB (LB-DEX) or GASW-Tris (GASW-DEX)[47] (Fisher Scientific; Waltham, MA, USA). Bacterial cultures were washed with either ASW (GASW lacking glycerol, tryptone, or yeast extract) or phosphate buffered saline (PBS) for *Vibrio* and *E*. *coli* strains, respectively.

#### Plasmid construction

All of the plasmids that were used are listed in (S1 Table), and the DNA oligonucleotide primers are listed in (S2 Table). The plasmid pBU226 is a suicide vector used to create a clean deletion of the *vtpR* homolog in OCN008 except for the first and last 18 nucleotides. Genomic DNA from OCN008 was used as template for PCR with the primer pairs 008-vtpR-up-EcoRI-F and Vcor-vtpR-up-OEX-R and Vcor-vtpR-down-OEX-F and 008-vtpR-down-XbaI-R to amplify regions up- and downstream of *vtpR*, respectively. The resulting PCR product was cloned as an *Eco*R1/*Xba*I fragment into the same sites in pSW4426T to create pBU226. Unless otherwise stated, all suicide plasmids were screened using PCR and Sanger sequencing using the primer pair pSW4426T-MCS-F and pSW4426T-MCS-R to confirm successful cloning.

The plasmid pBU247 is a suicide vector used to create a clean deletion of the *vasK* homolog in OCN008 except for the first and last 18 nucleotides. OCN008 genomic DNA was used as template for PCR with the primer pairs 008vasK-up-EcoR1-F and 008vasK-up-OEX-R and 008vasK-down-OEX-F and 008vasK-down-XbaI-R. The resulting PCR product was cloned as an *Eco*R1/*Xba*I fragment into the same sites in pSW4426T to create pBU247.

The plasmid pBU266 is a suicide vector used to create a clean deletion of the *vtpA* homolog in OCN008 except for the first and last 18 nucleotides. OCN008 genomic DNA was used as template for PCR with the primer pairs vtpA-up-EcoRI-F and vtpA-up-OEX-R and vtpA-down-OEX-F and vtpA-down-SpeI-R. The resulting PCR product was cloned as an *Eco*R1/*Spe*I fragment into the same sites in pSW4426T to create pBU266.

The plasmid pBU267 is a suicide vector used to create a clean deletion of the *vtpB* homolog in OCN008 except for the first and last 18 nucleotides. OCN008 genomic DNA was used as template for PCR with the primer pairs vtpB-up-SpeI-F and vtpB-up-OEX-R and vtpB-down-OEX-F and vtpA-down-SpeI-R. The resulting PCR product was cloned as a *Spe*I fragment into the *Xba*I site in pSW4426T that had been previously dephosphorylated with FastAP Thermosensitive Alkaline Phosphatase (Thermo Fisher Scientific) to create pBU266.

The plasmid pBU270 is a replicative vector used to express a wild type copy of *vasK* to complement the OCN008 *vasK* mutant. OCN008 genomic DNA was used as template for PCR with the primer pair vasK-XbaI-F and vasK-XbaI-R. The resulting PCR product was cloned as an *Xba*I fragment into the same site in pBU246 that had been previously dephosphorylated to create pBU270.

The plasmid pBU271 is a replicative vector used to express a wild type copy of *vtpR* to complement the OCN008 *vtpR* mutant. OCN008 genomic DNA was used as template for PCR with the primer pair 008-vtpR-SacI-F and 008-vtpR-XbaI-R. The resulting PCR product was cloned as a *Sac*I/*Xba*I fragment into the same sites in pBU246 to create pBU271.

#### Mutant creation

All *V*. *coralliilyticus* suicide vectors were introduced using tri-parental conjugations with *E*. *coli* as previously described[55]. Donor and recipient strains were grown overnight with the appropriate antibiotics and DAP or DT as required (Sigma-Aldrich). Overnight cultures were diluted 1:1000 in fresh culture medium without antibiotics, grown to an optical density measured at 600 nm (OD_600_) of 0.4, and then one ml washed three times with either ASW or PBS for *Vibrio* or *E*. *coli* strains, respectively. The strains were then combined, resuspended in ASW to a total volume of 50 μl, and spotted onto GASW-DEX plates supplemented with DAP and DT. Conjugation spots were incubated at 30°C for 15 h before being resuspended in ASW, washed three times with ASW, diluted, and plated onto GASW-DEX supplemented with chloramphenicol, but lacking DAP or DT, at 27°C. Chloramphenicol-resistant colonies, were streaked for isolation on GASW-DEX with spectinomycin and streptomycin, the colonies were then screened for the presence of the suicide vector integrated into the chromosome using colony PCR and the primers pSW4499-cat-F and pSW4499-oriT-R. Colonies of *Vibrio* with the integrated plasmid were grown for 15 h in GASW-DEX broth. Cultures were washed with ASW three times, diluted, and plated onto GASW-ARA to isolate mutants with a clean deletion of the target gene. Mutants were confirmed using PCR and primers specific to the gene being mutated.

#### Challenge assays

Bacterial isolates were grown in liquid Zobell 2216E media overnight, washed, diluted 1:10 into fresh media, and grown for approximately 3 h. The cultures were then concentrated to an OD_600_ of 10 via centrifugation at 8,600 x g for 5 minutes. Predator and challenger were mixed 1:1 (v:v; 10 μl total) and 5 μl aliquots of the co–cultures were spotted onto Zobell 2216E agar. We note that the starting colony forming units (CFUs) for each *V*. *cholerae* strain was ~1.3x10^7^ mL^-1^ in the competition assays. Starting CFUs were not determined for the other isolates. Challenge assays and *V*. *cholerae* monocultures (controls) were incubated for 4 h at 25°C. The cells were then re-suspended, serial–diluted, and plated onto antibiotic selection media to recover and enumerate the surviving predator and challenger. Each challenge was independently repeated three times (biological replicates, *n* = 3), and each biological replicate consisted of three technical replicates. To ensure that our results were consistent and that the data interpretation was standardized, each bacterial challenger was screened against the same batch-culture of the predator. Statistical difference between the mean +/- SD of treatments was determined by two-tailed t-tests using GraphPad Prism version 7.0 (GraphPad Software, Inc.) and is described within each figure legend.

#### Biofilm assays

The ability of the *V*. *coralliilyticus* strains to produce a biofilm was measured using a modified crystal violet assay[56]. Cultures of *V*. *coralliilyticus* were initially grown overnight (approximately 15 h) in GASW-Tris. The cultures were then diluted 1:1000 into fresh media. In a 24-well plate, one ml aliquots of the diluted cultures were placed to each well (four replicates per strain). The plates were then incubated in a humidified incubator at 28°C for 48 h. After incubation, the liquid cultures were then carefully aspirated using a pipette while being careful not to disrupt the sides of the wells. One ml of ASW was then added to each well and then removed via a pipette. This washing process was repeated two more times. Into each well, one ml of a 0.1% crystal violet solution was then added and incubated at room temperature for 15 min. The crystal violet solution was then poured off and the wells were again washed three times with ASW. The plates were then dried overnight with their lids off and inverted. After drying, one ml of a 30% acetic acid solution was added to each well, incubated at room temperature for 15 min, and then 500 μl of each well was transferred to a new 24-well plate. The absorbance of each well was measured at 550 nm in a plate reader (Epoch Microplate Spectrophotometer). One ml aliquots of sterile GASW-Tris processed in an identical manner as the bacterial cultures served as the blank.

## Results & discussion

### Select Ɣ-Proteobacteria exhibited susceptibility to T6SS attack by *V*. *cholerae*

**Fig 1:**
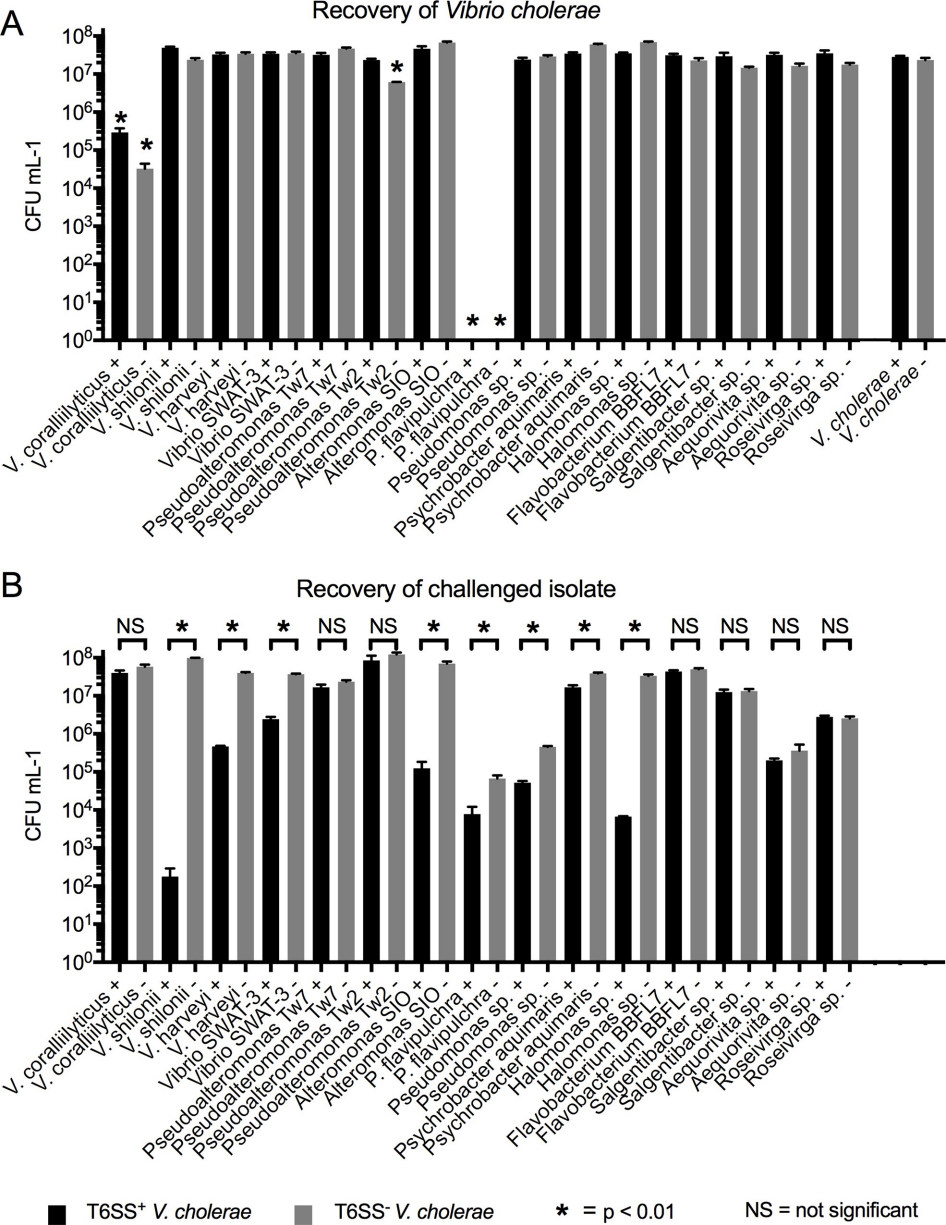
Summary of *V*. *cholerae* challenge assays. T6SS^+^ *V*. *cholerae* (black bars) or T6SS^-^ *V*. *cholerae* (grey bars) were challenged against marine bacterial isolates in competition assays. *V*. *cholerae* strains were also grown in monoculture to serve as controls. Error bars represent the mean ±SD of three biological replicates. (A) Recovered CFUs mL^-1^ after each challenge assay are shown side–by–side for T6SS^+^ and T6SS^-^ *V*. *cholerae*. Asterisks denote statistically significant differences (two-tailed t-test) between the CFUs recovered for the indicated *V*. *cholerae* strain after the challenge assay in comparison to its recovery when grown in monoculture (far right of graph). (B) Brackets indicate a two-tailed t-test implemented to determine statistically significant differences between recovered CFUs mL^-1^ for each challenged isolate after its competition assay against either T6SS^+^ or T6SS^-^ *V*. *cholerae*.

To test the efficacy of *V*. *cholerae* T6SS deployment against marine bacteria, we challenged a suite of marine isolates from different environmental and phylogenetic backgrounds (Table 2) against *V*. *cholerae* with an active T6SS (T6SS^+^), or its isogenic T6SS knockout mutant (T6SS^-^) derivative that was created and confirmed in a previous study (see Table 1). Colony forming unit recoveries were reduced by ~90% for eight out of the 15 isolates in the challenges against T6SS^+^ *V*. *cholerae* in comparison to the challenges against T6SS^-^ *V*. *cholerae* (p < 0.01, two-tailed t-test), indicating that those isolates were susceptible to T6SS attack (Fig 1B). The eight susceptible isolates were all Ɣ-Proteobacteria, including three members of the order Vibrionales (*V*. *harveyi*, *Vibrio* sp. SWAT-3, and *V*. *shilonii*), a bacterial group that has been previously reported to contain marine species that are sensitive to *V*. *cholerae*’s T6SS[39]. The other susceptible isolates were from the orders Alteromonadales (*Alteromonas* Alt-SIO and *Pseudoalteromonas flavipulchra*), Oceanospirillales (*Halomonas* sp. 73), and Pseudomonadales (*Pseudomonas sp*. and *Psychrobacter aquimaris*). To our knowledge, this is the first report of marine bacterial susceptibility to a T6SS from the three aforementioned orders.

Surprisingly, four of the isolates were killed or inhibited when challenged against both T6SS^+^ and T6SS^-^ *V*. *cholerae*. These isolates were from the phyla Bacteroidetes (*Aequorivita* sp. 97 and *Roseivirga* sp. 121) and Ɣ-Proteobacteria (*P*. *flavipulchra* and *Pseudomonoas* sp. 2) (Fig 1B). Importantly, we note that these strains appear to have suffered no mortality when grown in monoculture under identical conditions, and that the monoculture recoveries were ~90% higher for each of the four strains in comparison to their recovery after the challenge against T6SS^+^ or T6SS^-^ *V*. *cholerae* (p < 0.01, two-tailed t-test; S1 Fig). While the mechanisms that led to their significant CFU reduction when co-cultured with *V*. *cholerae* were not further explored here, we offer several scenarios that might explain our observations: (1) the challenged isolates grew slower in co-culture, (2) the challenged isolates were outcompeted for resources, and/or (3) the challenged isolates were killed, either by toxic byproducts of metabolism or an alternative inhibitory mechanism used by *V*. *cholerae*.

Interestingly, we also observed that when T6SS^+^ and T6SS^-^ *V*. *cholerae* were each challenged against *Pseudoalteromonas flavipulchra* it resulted in death or inhibition for both *V*. *cholerae* strains (no CFUs were recovered, Fig 1A). These results may be explained in part by *P*. *flavipulchra*’s highly antagonistic nature which has been demonstrated to inhibit the growth of a number of marine bacteria via the release of inhibitory chemical(s) [10]. This species is also known to produce L-amino acid (lysine or glycine) oxidases that are capable of hydrolyzing amino acids present within cells or in the growth media to produce hydrogen peroxide [57]. These enzymes are bactericidal to a wide range of isolates and can be autotoxic[58–60]. Although *P*. *flavipulchra* achieved a CFU recovery of ~1.5 x 10^7^ mL^-1^ after 4 h monoculture incubation (S1 Fig), it is possible that production of these toxic compounds could be triggered by the presence of *V*. *cholerae* when grown in co-culture. Such scenarios, in combination, or with any exacerbating effects that *V*. *cholerae* may exert in the co-culture, could explain the observed loss of CFUs for both competing species.

### Several isolates were resistant to *V*. *cholerae*’s T6SS

Four out of the 15 challengers co-existed with *V*. *cholerae*, as these isolates went unaffected by *V*. *cholerae*’s T6SS (Fig 1B) and did not kill either T6SS^+^ or T6SS^-^ *V*. *cholerae* in their respective assays (Fig 1A). Two of the co-existing isolates were from the phylum Bacteroidetes (*Flavobacteria* sp. BBFL7 and *Salgentibacter* sp. 1). Interestingly, some members of the Bacteroidetes have been shown to exhibit immunity against T6SS effector proteins [35]. This is relevant to marine microbial ecology since Bacteroidetes are commonly found as the predominant taxa on bacteria–rich marine particles [61]. Resistance to contact–dependent killing mechanisms, such as the T6SS, may help enable these taxa to colonize and proliferate in such environments. The other isolates that we found to co-exist with T6SS^+^ *V*. *cholerae* were two closely related Ɣ-Proteobacteria (*Pseudoalteromonas* Tw7 and *Pseudoalteromonas* Tw2), which were evidently resistant or immune to *V*. *cholerae*’s T6SS (Fig 1B). It is also noteworthy that CFU recovery for T6SS^-^ *V*. *cholerae* was significantly reduced after the competition with *Pseudoalteromonas* Tw2 in comparison to the recovery of T6SS^+^ *V*. *cholerae* (p < 0.01, two-tailed t-test; Fig 1A). Overall, of the 15 isolates tested, only *V*. *coralliilyticus* displayed the ability to resist *V*. *cholerae’s* T6SS (Fig 1B) and to kill both T6SS^+^ and T6SS^-^ *V*. *cholerae* (p < 0.01, two-tailed t-test; Fig 1A) (further discussed below).

Collectively, our results demonstrate that marine bacteria from a range of different taxa were susceptible to the T6SS of *V*. *cholerae* 2740–80, and that conversely, a number of taxa were resistant to its T6SS. We have begun looking into the resistance mechanisms that were employed by the isolates in our study, starting with *V*. *coralliilyticus*, an important coral and oyster pathogen [46, 48, 62–65]. It has been suggested that *V*. *coralliilyticus* is capable of altering a susceptible coral’s microflora that is thought to protect their host from infection [66], however, no mechanisms have been proposed for how the pathogen accomplishes this. Similarly, *V*. *coralliilyticus* is able to dominate the bacterial communities within shellfish hatcheries, suggesting an effective mechanism for competition [67]. These observations could be explained in part by this pathogen’s ability to defend itself against other bacteria, or to kill other bacteria, as we found in the challenge against *V*. *cholerae*.

#### *V*. *coralliilyticus* evidently killed *V*. *cholerae* by using its own T6SS

**Fig 2:**
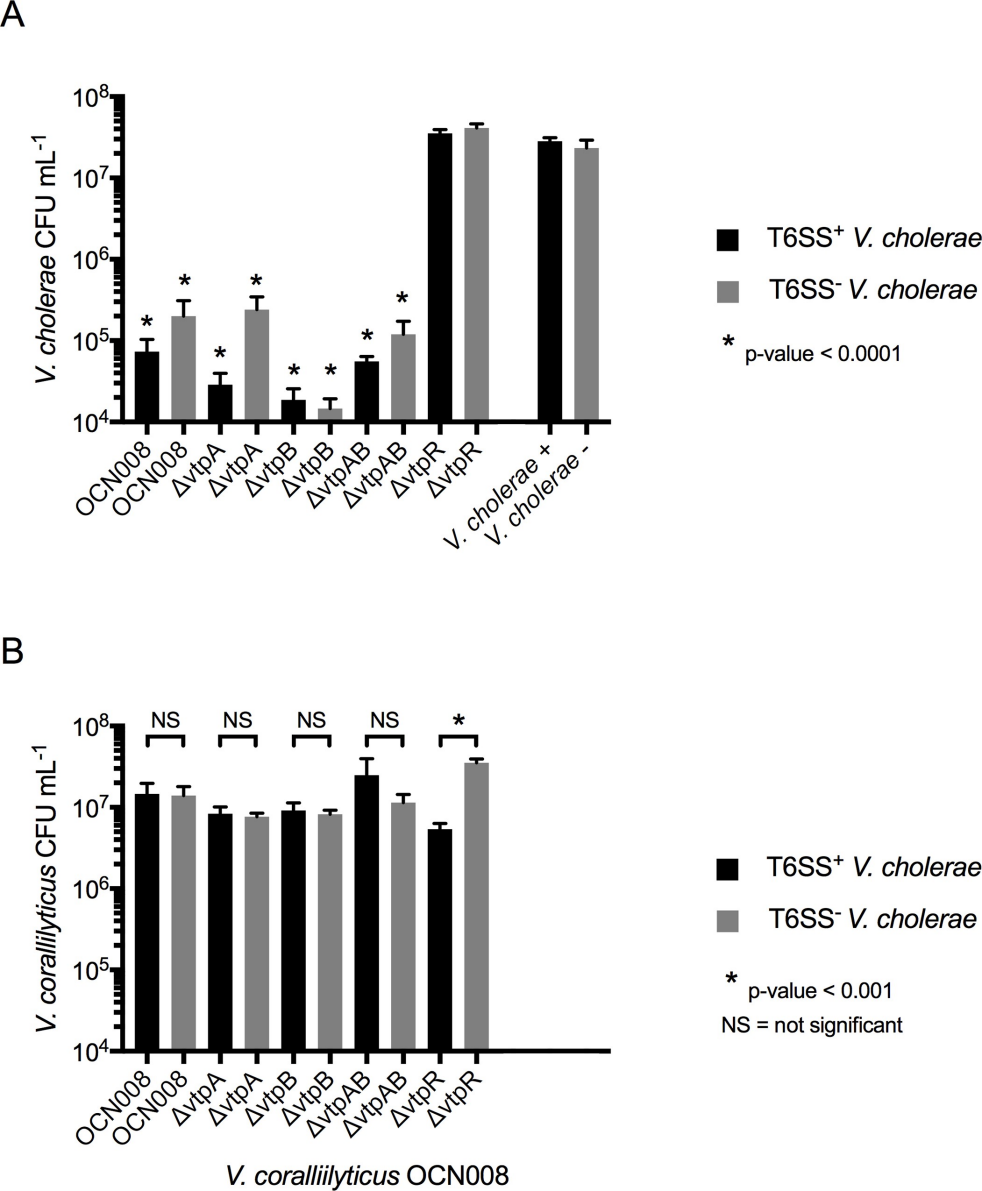
*V*. *coralliilyticus* protease-mutant challenge assays. *V*. *coralliilyticus* OCN008, protease-mutant derivatives (*ΔvtpA*, *ΔvtpB*, and *ΔvtpAB*), and the *ΔvtpR* mutant were challenged against T6SS^+^ *V*. *cholerae* (black bars) or T6SS^-^ *V*. *cholerae* (grey bars). *V*. *cholerae* strains were also grown in monoculture to serve as controls. Error bars represent the mean ±SD of three biological replicates. (A) *V*. *cholerae* CFU recovery. Asterisks denote statistically significant differences (two-tailed t-test) between the CFUs recovered for the indicated *V*. *cholerae* strain after the challenge assay in comparison to its recovery when grown in monoculture (far right of graph). (B) Brackets indicate a two-tailed t-test implemented to determine statistically significant differences between recovered CFUs mL^-1^ for each tested *V*. *coralliilyticus* isolate after its competition assay against either T6SS^+^ or T6SS^-^ *V*. *cholerae*.

We hypothesized that *V*. *coralliilyticus* may have (1) been intrinsically resistant to the T6SS attack by *V*. *cholerae*, (2) killed *V*. *cholerae* before itself was attacked, or (3) survived due to a combination of both scenarios. For the first hypothesis, we considered that the well-characterized proteolytic activity of *V*. *coralliilyticus* [63, 65, 68–70] might provide resistance to *V*. *cholerae*’s T6SS by degrading the T6SS apparatus or effector proteins, or by killing *V*. *cholerae* directly. To test this, knockout mutants were created using *V*. *coralliilyticus* strain OCN008 which had clean deletions of the quorum sensing regulator *vtpR*, which regulates protease activity in this species [71], as well as the protease-encoding genes *vtpA* and *vtpB* individually and in combination (*vtpAB*). These four mutant strains were challenged against T6SS^+^ and T6SS^-^ *V*. *cholerae* in competition assays. We found that the Δ*vtpR* mutant recovery was reduced by nearly one log-fold in the challenge against T6SS^+^ *V*. *cholerae* in comparison to the challenge against T6SS^-^ *V*. *cholerae* (p < 0.001, two-tailed t-test, Fig 2B) and that it was unable to kill *V*. *cholerae* (Fig 2A). However, all three *V*. *coralliilyticus* protease mutants (Δ*vtpA*, Δ*vtpB*, and Δ*vtpAB*) resisted *V*. *cholerae*’s T6SS (Fig 2B) and retained their ability to kill *V*. *cholerae* at levels equivalent to the wild-type strain (p < 0.0001, two-tailed t-test; Fig 2A). Therefore, we concluded that while VtpR did play a role in the success of *V*. *coralliilyticus* survival against T6SS^+^ *V*. *cholerae*, the tested proteases were unlikely to be the mechanism that protected *V*. *coralliilyticus* from T6SS attack and were not responsible for the observed killing of *V*. *cholerae*.

As a homolog of the *V*. *cholerae* quorum sensing regulator HapR, VtpR is believed to regulate a wide range of physiological functions [71]. Recently, Strutzmann and Blokesch (2016) reported that mutations that inactivated HapR resulted in reduced T6SS activity for *V*. *cholerae* [72]. We considered that if *V*. *coralliilyticus* carried a functional T6SS that was regulated in part by VtpR, then the deletion of *vtpR* in OCN008 may have diminished or eliminated T6SS expression in our experiments, explaining our observation that the Δ*vtpR* strain was unable to kill *V*. *cholerae*. Our hypothesis that *V*. *coralliilyticus* had a T6SS was partially supported by Kimes et al. (2011) who previously observed needle-like structures within *V*. *coralliilyticus* and found upregulated expression of predicted T6SS-associated proteins at temperatures that correlated with increased virulence [73]. However, leading up to this study it was unknown if *V*. *coralliilyticus* had a functional T6SS that could kill microbial prey.

**Fig 3:**
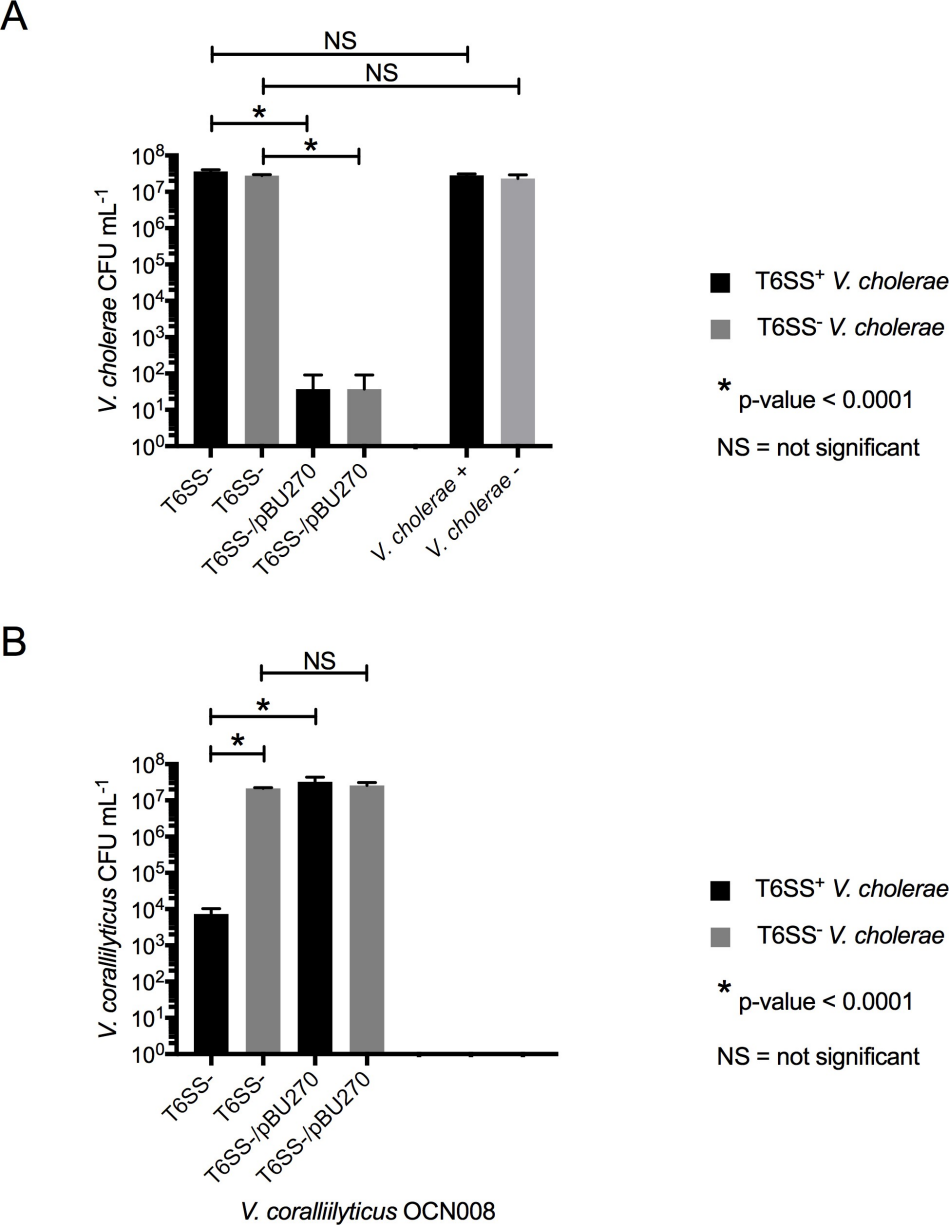
Genetically complemented *V*. *coralliilyticus* T6SS mutant challenge assay. The *V*. *coralliilyticus* OCN008 Δ*vasK* strain (T6SS^-^) and the Δ*vasK* strain carrying a plasmid expressing a wild-type copy of *vasK* (pBU270) were challenged against T6SS^+^ *V*. *cholerae* (black bars) or T6SS^+^ *V*. *cholerae* (grey bars). *V*. *cholerae* strains were also grown in monoculture to serve as controls. Error bars represent the mean ±SD of three biological replicates. (a) *V*. *cholerae* CFU recovery. Recovered CFUs mL^-1^ for *V*. *cholerae* strains after the challenge against T6SS^-^ *V*. *coralliilyticus* were compared to their respective recovery when grown in monoculture (far right of graph) or to their recovery after the challenge against T6SS^-^/pBU270 *V*. *coralliilyticus*. (b) *V*. *coralliilyticus* CFU recovery. Recovered CFUs for T6SS^-^ *V*. *coralliilyticus* after the challenge against T6SS^+^ *V*. *cholerae* was compared to the recovered CFUs when challenged against T6SS^-^ *V*. *cholerae*. Recovered CFUs for T6SS^-^ *V*. *coralliilyticus* after the challenge against T6SS^+^ or T6SS^-^ *V*. *cholerae* was also compared to the recovery of T6SS^-^/pBU270 *V*. *coralliilyticus* when challenged against T6SS^+^ or T6SS^-^ *V*. *cholerae*. Brackets indicate a two-tailed t-test.

To investigate if *V*. *coralliilyticus* was using a T6SS in the competition assays, we created a *V*. *coralliilyticus vasK* deletion mutant (T6SS^-^) and challenged it against T6SS^+^ or T6SS^-^ *V*. *cholerae*. The Δ*vasK* mutant had a deletion in a gene predicted to encode a homolog of VasK, which is essential for *V*. *cholerae* T6SS function [28]. In accordance with our hypothesis, we found that both T6SS^+^ and T6SS^-^ *V*. *cholerae* survived the challenge assays (Fig 3A), demonstrating that the killing of *V*. *cholerae* by *V*. *coralliilyticus* did require the *vasK* gene. Moreover, the T6SS^-^ *V*. *coralliilyticus* mutant was susceptible to T6SS attack by *V*. *cholerae* (p < 0.0001, two-tailed t-test; Fig 3b), demonstrating that *V*. *coralliilyticus* was not inherently resistant to the *V*. *cholerae* T6SS. Genetic complementation of the T6SS^-^ *V*. *coralliilyticus* strain restored the mutants’ ability to kill *V*. *cholerae* (p < 0.0001, two-tailed t-test; Fig 3A) and its apparent resistance to T6SS-mediated attack (Fig 3B). Thus, the inability of the T6SS^-^ *V*. *coralliilyticus* strain to kill *V*. *cholerae*, in combination with the mutant’s susceptibility to T6SS-mediated killing by *V*. *cholerae*, strongly suggests that *V*. *coralliilyticus* used its own T6SS to kill *V*. *cholerae* in the challenge. Therefore, *V*. *coralliilyticus* evidently survived by winning in the ‘quick draw’, or by striking more effectively, as opposed to being resistant to attack. This contrasts with the previously described tit-for-tat interactions between *V*. *cholerae* and *P*. *aeruginosa*, in which, *P*. *aeruginosa* is described as intrinsically resistant to *V*. *cholerae* T6SS-mediated killing, while utilizing its own T6SS only in response to bacterial aggression [38]. Furthermore, given that the T6SS^-^ *V*. *coralliilyticus* mutant was found to be susceptible to *V*. *cholerae*’s T6SS, we were able to rule out the hypothesis that *V*. *coralliilyticus* employed other natural resistance mechanisms such as immunity to the toxic effector proteins or protective exopolysaccharide (EPS) “armor” that have been previously described [35, 36]. Interestingly, the Δ*vtpR* strain, which was susceptible to T6SS^+^ *V*. *cholerae*, was found to produce more EPS in comparison to the wild-type strain (p < 0.0001, Tukey’s multiple comparisons test; S2 Fig) further suggesting that EPS production was not the protective mechanism for *V*. *coralliilyticus* in our study. In all, these results are the first evidence for a functional *V*. *coralliilyticus* T6SS, which we found to be effective at killing *V*. *cholerae* and required for resistance to T6SS-mediated killing by *V*. *cholerae*.

#### *V*. *coralliilyticus* T6SS has implications for coral and shellfish health

**Fig 4:**
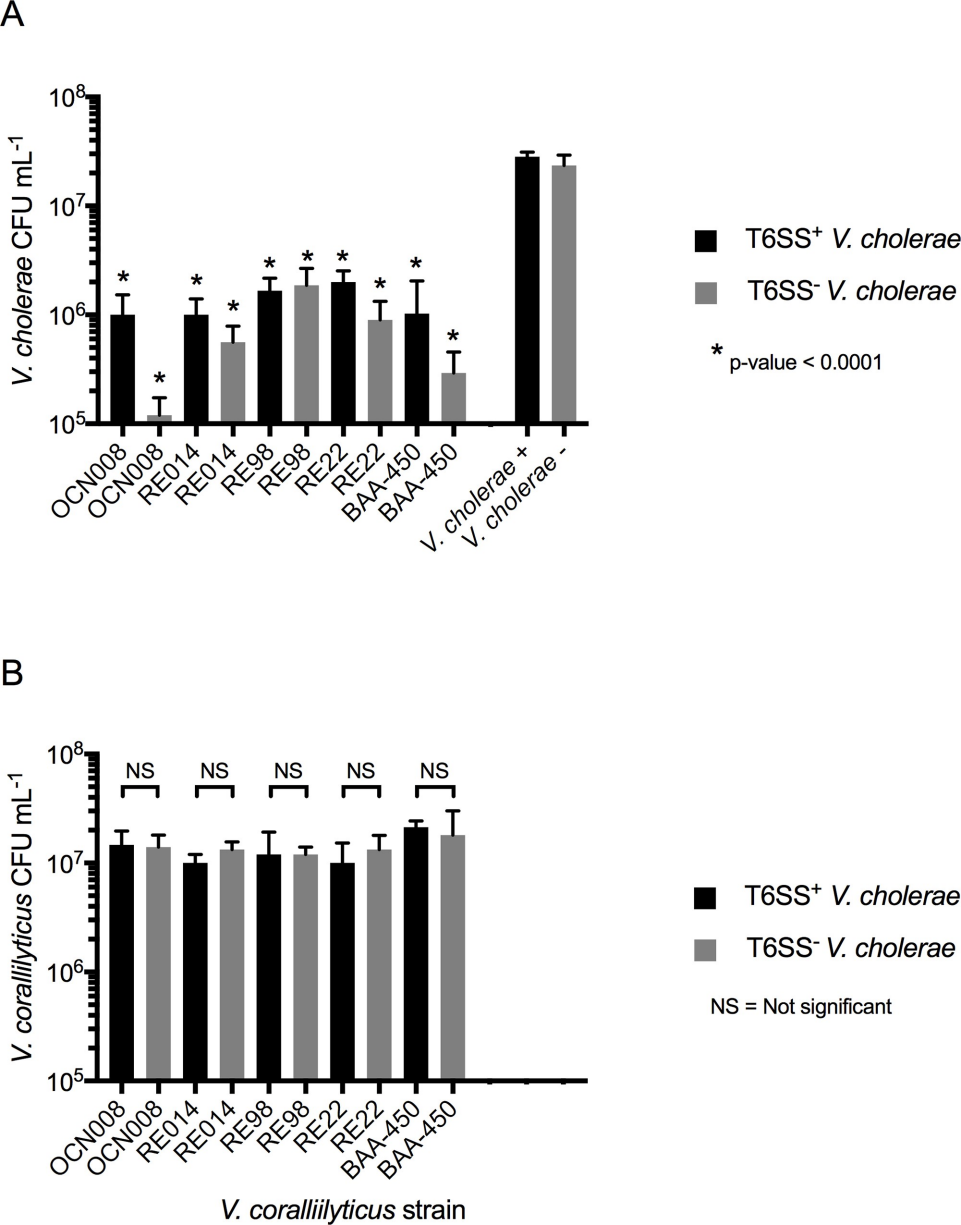
*V*. *coralliilyticus* challenge assays. Five *V*. *coralliilyticus* strains (OCN008, RE014, RE98, RE22, and BAA-450) were challenged against T6SS^+^ *V*. *cholerae* (black bars) and T6SS^-^ *V*. *cholerae* (grey bars). *V*. *cholerae* strains were also grown in monoculture to serve as controls. Error bars represent the mean ±SD of three biological replicates. (a) *V*. *cholerae* CFU recovery. Asterisks denote statistically significant differences (two-tailed t-test) between the CFUs recovered for the indicated *V*. *cholerae* strain after the challenge assay in comparison to its recovery when grown in monoculture (far right of graph). (b) *V*. *coralliilyticus* CFU recovery. Brackets indicate a two-tailed t-test implemented to determine statistically significant differences between recovered CFUs mL^-1^ for each tested *V*. *coralliilyticus* isolate after its competition assay with either T6SS^+^ or T6SS^-^ *V*. *cholerae*.

Intrigued by the ability of *V*. *coralliilyticus* OCN008 to resist *V*. *cholerae*’s T6SS and to kill *V*. *cholerae* with its own T6SS, we conducted further experiments to determine if these characteristics were strain-specific. Four additional *V*. *coralliilyticus* strains including known coral and shellfish pathogens (OCN014, RE98, RE22, and BAA-450; Table 1) were challenged against T6SS^+^ and T6SS^-^ *V*. *cholerae*. Consistent with our initial result, we found that all four of the *V*. *coralliilyticus* strains were not affected by T6SS^+^ *V*. *cholerae* (Fig 4B) and that the survival of both T6SS^+^ and T6SS^-^ *V*. *cholerae* was reduced by > 99% (p < 0.0001, two-tailed t-test) by all of the tested *V*. *coralliilyticus* strains including OCN008, which was run alongside them (Fig 4A). This suggested the presence of conserved mechanisms shared between the strains that offered protection to *V*. *coralliilyticus* and enabled each of the strains to kill *V*. *cholerae*. We surmise that the four *V*. *coralliilyticus* strains (OCN014, RE22, RE98, and BAA-450) that killed T6SS^+^ and T6SS^-^ *V*. *cholerae* (Fig 4A), like strain OCN008, carried a functional T6SS. While not explicitly tested here, that hypothesis is supported by predicted protein homology. Proteins required for T6SS functionality in *V*. *cholerae* shared 24–69% amino acid homology with predicted proteins in strain OCN008 (Table 3) and these proteins were present in the four other *V*. *coralliilyticus* strains (sharing 99–100% amino acid homology; Table 4).

**Table 3:** Comparison of *V*. *coralliilyticus* T6SS-associated proteins to select *V*. *cholerae* proteins.

*V*. *cholerae* T6SS protein	OCN008 homolog	OCN014 homolog	BAA-450 homolog	RE22 homolog	RE98 homolog	General protein function
**VasA (VCA0110)**	**ERB64088 (43%)**	**AIS57250 (43%)**	**EEX32046 (43%)**	**KPH23943 (43%)**	**AIW21233 (43%)**	**Structural**
**VasK (VCA0120)**	**MH794511 (24%)**	**AIS57248 (24%)**	**WP_039952112 (24%)**	**KPH23940 (24%)**	**AIW21236 (24%)**	**Structural**
**VipA (VCA0107)**	**ERB64085 (55%)**	**AIS57253 (55%)**	**EEX32049 (55%)**	**KPH23946 (55%)**	**AIW21230 (55%)**	**Structural**
**VipB (VCA0108)**	**ERB64086 (69%)**	**AIS57252 (69%)**	**EEX32048 (69%)**	**KPH23945 (69%)**	**AIW21231 (69%)**	**Structural**
Hcp-1* **(VC1415)**	**ERB62208 (55%)**	**AIS57260 (55%)**	**EEX32057 (55%)**	**KPH23954 (55%)**	**AIW21222 (55%)**	**Structural**
Hcp-2* **(VCA0017)**	**ERB62208 (55%)**	**AIS57260 (55%)**	**EEX32057 (55%)**	**KPH23954 (55%)**	**AIW21222 (55%)**	**Structural**
**VasH (VCA0117)**	**ERB65234 (42%)**	**AIS57262 (42%)**	**EEX32059 (42%)**	**KPH23956 (42%)**	**AIW21220 (42%)**	**σ**^**54**^ **activator**
**VasF (VCA0115)**	**ERB64099 (37%)**	**AIS57243 (37%)**	**EEX32037 (37%)**	**KPH23935 (37%)**	**AIW21241 (37%)**	**Effector translocation**
VgrG-2* **(VCA0018)**	**ERB64077 (34%)**	**AIS57259 (34%)**	**EEX32056 (34%)**	**KPH23953 (34%)**	**AIW21223 (34%)**	**Effector**
VgrG-3* **(VCA0123)**	**ERB64077 (32%)**	**AIS57259 (32%)**	**EEX32056 (32%)**	**KPH23953 (32%)**	**AIW21223 (32%)**	**Effector**

*More than one *V*. *cholerae* homolog is most similar to multiple homologs in *V*. *coralliilyticus*

**Table 4:** Comparison of *V*. *coralliilyticus* T6SS-related proteins to OCN008 proteins.

OCN008 protein	OCN014 homolog	BAA-450 homolog	RE22 homolog	RE98 homolog
**VasA (ERB64088)**	**AIS57250 (99%)**	**EEX32046 (100%)**	**KPH23943 (99%)**	**AIW21233 (100%)**
**VasK (MH794511)**	**AIS57248 (99%)**	**WP_039952112 (99%)**	**KPH23940 (99%)**	**AIW21236 (99%)**
**VipA (ERB64085)**	**AIS57253 (100%)**	**EEX32049 (100%)**	**KPH23946 (100%)**	**AIW21230 (99%)**
**VipB (ERB64086)**	**AIS57252 (100%)**	**EEX32048 (100%)**	**KPH23945 (100%)**	**AIW21231 (99%)**
**Hcp (ERB62208)**	**AIS57260 (100%)**	**EEX32057 (100%)**	**KPH23954 (100%)**	**AIW21222 (100%)**
**VasH (ERB65234)**	**AIS57262 (99%)**	**EEX32059 (99%)**	**KPH23956 (99%)**	**AIW21220 (99%)**
**VasF (ERB64099)**	**AIS57243 (100%)**	**EEX32037 (100%)**	**KPH23935 (100%)**	**AIW21241 (100%)**
**VgrG (ERB64077)**	**AIS57259 (100%)**	**EEX32056 (99%)**	**KPH23953 (99%)**	**AIW21223 (99%)**

These results could have a large impact on the understanding and treatment of coral and shellfish health. For example, strains OCN008, OCN014, and BAA-450 have been described as etiological agents of disease for multiple genera of coral [46, 63, 65], and strains RE98 and RE22 have been implicated in mass shellfish larvae mortalities [48, 62, 64]. We propose that the T6SS of *V*. *coralliilyticus* could be an important mechanism for the displacement of, and protection against host-associated bacteria, as it attempts to colonize potential hosts. In contrast to the displacement of the host microflora, *V*. *coralliilyticus* might also use the T6SS to attack other host-associated organisms or the host’s cells directly. Studies have suggested that during infections some strains of *V*. *coralliilyticus* kill the photosynthetic algal symbionts within coral cells (*Symbiodinium* spp.), resulting in coral bleaching [65, 68]. Given that the *V*. *cholerae* T6SS has been shown to kill eukaryotic organisms, such as the amoeba *Dictyostelium discoideum* [14], it is tempting to speculate that the *V*. *coralliilyticus* T6SS may be capable of killing *Symbiodinium*. Moreover, while it is still unclear if *V*. *coralliilyticus* acts as an intracellular pathogen, a recent study has demonstrated that *V*. *coralliilyticus* can end up within coral cells and vesicles during infection [74]. It might be possible for *V*. *coralliilyticus* to respond in these environments with its T6SS to escape host vesicles, similar to how *V*. *cholerae* defends against predation by *D*. *discoideum* phagocytosis. Such mechanisms have been reported for the intracellular pathogens *Francisella tularensis* and *Burkholderia pseudomallei*, which are able to use their T6SS to escape vesicles and macrophages, or to spread from cell to cell [75, 76]. We suggest that further investigations are warranted to better understand the role of T6SS deployment by *V*. *coralliilyticus* in coral and shellfish pathogenesis, which may aid in the protection of these environmentally and economically-important organisms.

## Conclusion

The bacterial type VI secretion system is present in various gram-negative bacteria and is capable of killing microbial prey. Here, we challenged a diverse set of marine bacterial isolates against T6SS^+^ *V*. *cholerae* and found high mortality rates for select members of the genus *Vibrio*. We also provided the first documentation for T6SS-mediated killing of marine Alteromonadales, Oceanospirillales, and Pseudomonadales. Additionally, a number of isolates from the Bacteroidetes and Ɣ-Proteobacteria phyla were found to be resistant to *V*. *cholerae*’s T6SS, including the important marine pathogen, *V*. *coralliilyticus*. All five of the tested *V*. *coralliilyticus* strains killed *V*. *cholerae*, presumably via the use of their own T6SS. We propose that bacterial susceptibility and resistance to contact-dependent killing mechanisms, such as the T6SS, might be important for the structuring of marine microbial communities in high bacterial density environments. Future work will be required to test the ecological impacts of such mechanisms *in situ* which should be possible by using a molecular-based approach in combination with direct imaging techniques.

## Supporting information

S1 Fig Select marine isolates grown in monoculture. Colony forming unit (CFU) recoveries of several marine isolates challenged against T6SS^+^ *V*. *cholerae* or T6SS^-^ *V*. *cholerae* (from Fig 1B) are compared to the isolates’ recovery when grown in monoculture (two-tailed t-test). The monocultures were grown as a follow–up experiment on separate days from the challenge assays but conducted in accordance with the same protocol that was used for the challenge assays.
(TIFF)

S2 Fig *V*. *coralliilyticus* OCN008 biofilm assay. A crystal violet assay was conducted to measure the amount of biofilm produced by wild type *V*. *coralliilyticus* and the Δ*vtpR* and Δ*vasK* strains. Blank = marine broth with no *V*. *coralliilyticus* cells. Higher optical density (OD) values at 550nm indicates more biofilm (extracellular polysaccharide) production. Statistical differences between treatments are denoted by different letters (ordinary one–way ANOVA, α = 0.05; p < 0.0001, Tukey’s multiple comparison test, a, b, c).
(TIFF)

S1 Table Conjugation strains and plasmids used in this study. *Abbreviations: Sm^R^ = resistant to streptomycin, Rf^R^ = resistant to rifampicin, Km^R^ = resistant to kanamycin, Em^R^ = resistant to erythromycin, Tc^R^ = resistant to tetracycline.
(DOCX)

S2 Table DNA oligonucleotide primers used in this study. (DOCX)
