# Supplementary figures and images for: Insight into the resilience and susceptibility of marine bacteria to T6SS attack by Vibrio cholerae and Vibrio coralliilyticus
Source: PLoS One. 2020 Jan 28;15(1):e0227864. doi: 10.1371/journal.pone.0227864 (PMC6986712; doi:10.1371/journal.pone.0227864)

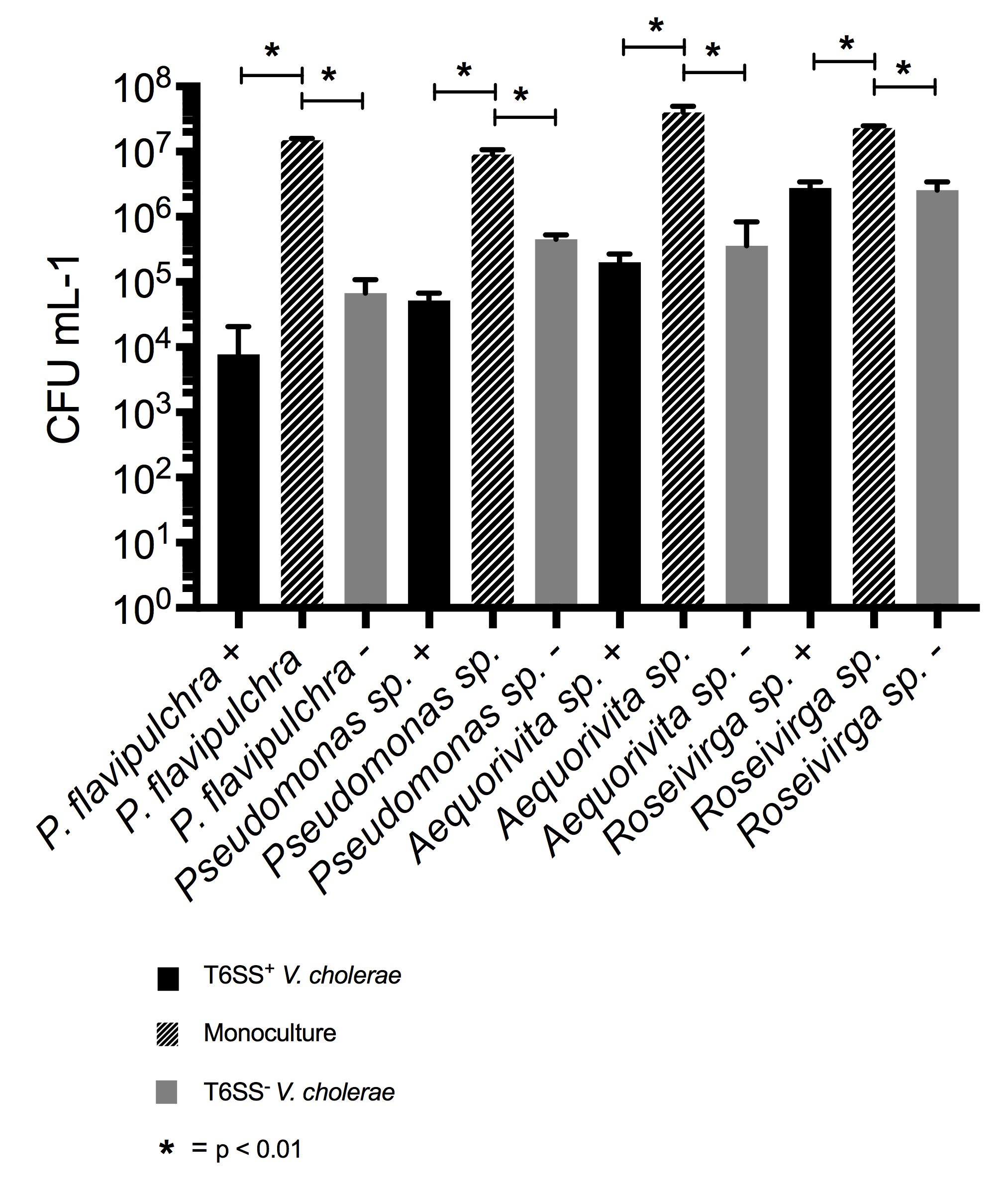

Supplement: S1 Fig — Colony forming unit (CFU) recoveries of several marine isolates challenged against T6SS+ V. cholerae or T6SS- V. cholerae (from Fig 1B) are compared to the isolates’ recovery when grown in monoculture (two-tailed t-test). The monocultures were grown as a follow–up experiment on separate days from the challenge assays but conducted in accordance with the same protocol that was used for the challenge assays. (TIFF) [file pone.0227864.s001.tiff]

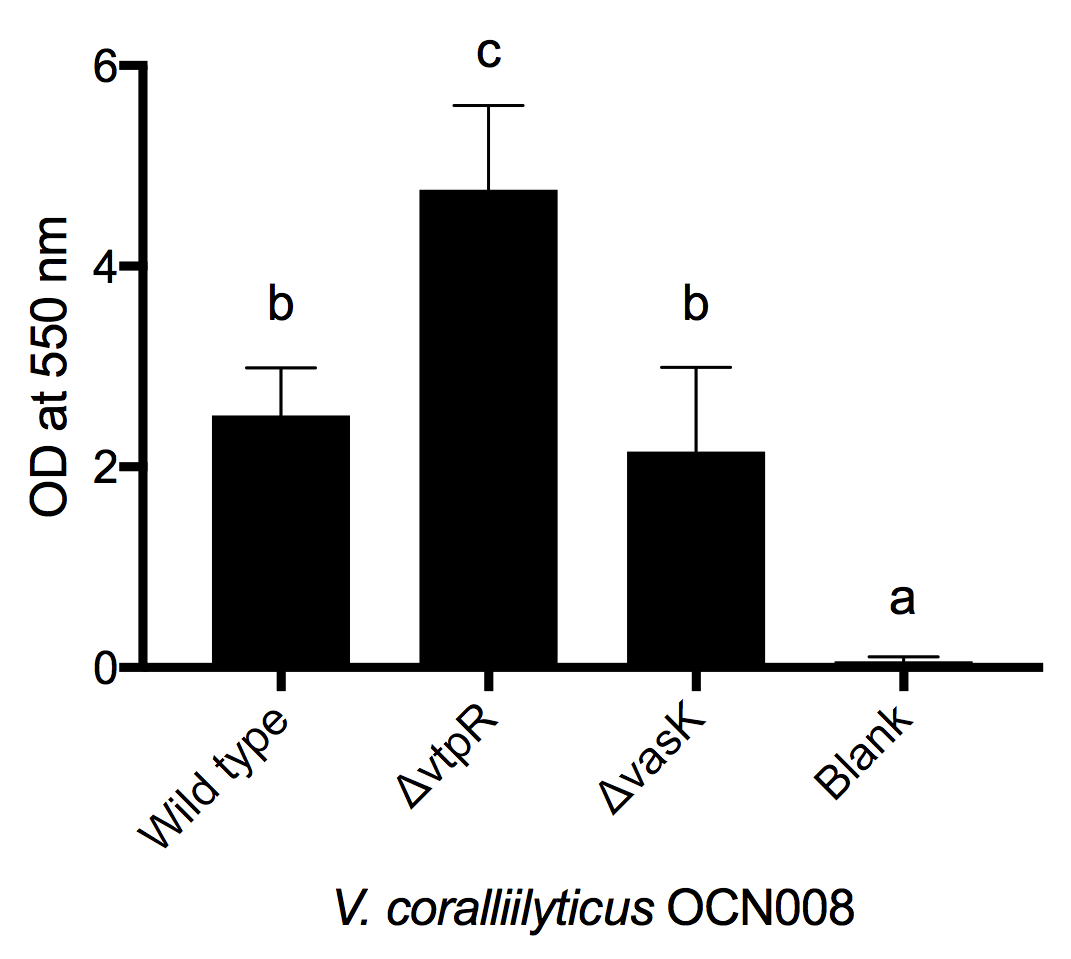

Supplement: S2 Fig — A crystal violet assay was conducted to measure the amount of biofilm produced by wild type V. coralliilyticus and the ΔvtpR and ΔvasK strains. Blank = marine broth with no V. coralliilyticus cells. Higher optical density (OD) values at 550nm indicates more biofilm (extracellular polysaccharide) production. Statistical differences between treatments are denoted by different letters (ordinary one–way ANOVA, α = 0.05; p < 0.0001, Tukey’s multiple comparison test, a, b, c). (TIFF) [file pone.0227864.s002.tiff]
