# Supplementary material for: Insight into the resilience and susceptibility of marine bacteria to T6SS attack by Vibrio cholerae and Vibrio coralliilyticus
Source: PLoS One. 2020 Jan 28;15(1):e0227864. doi: 10.1371/journal.pone.0227864 (PMC6986712; doi:10.1371/journal.pone.0227864)
Supplement: S1 Table — *Abbreviations: SmR = resistant to streptomycin, RfR = resistant to rifampicin, KmR = resistant to kanamycin, EmR = resistant to erythromycin, TcR = resistant to tetracycline. (DOCX) [file pone.0227864.s003.docx]

| **ID** | **Description** | **Citation or source** |
| --- | --- | --- |
| *E. coli* β3916 | Conjugation strain; Δ*dapA*::(*erm*-*pir*); Km^R^, Em^R^, Tc^R^ | (Le Roux et al. 2007)^75^ |
| *E. coli* π3813 | Conjugation strain; Δ*thyA*::(*erm*-*pir*); Em^R^ | (Le Roux et al. 2007)^75^ |
| pSW4436T | Base plasmid for suicide vectors that carries an inducible *ccdB* selectable marker; Cm^R^, Sp^R^, Sm^R^ | (Le Roux et al. 2007)^75^ |
| pBU226 | Suicide vector used to delete the *vtpR* homolog in OCN008; Cm^R^, Sp^R^, Sm^R^ | This study |
| pBU246 | Base plasmid for inducible expression plasmids used to complement *V. coralliilyticus* deletion mutants; Cm^R^, Sp^R^, Sm^R^ | (Ushijima et al. 2018)^48^ |
| pBU247 | Suicide vector used to delete the *vasK* homolog in OCN008; Cm^R^, Sp^R^, Sm^R^ | This study |
| pBU266 | Suicide vector used to delete the *vtpA* homolog in OCN008; Cm^R^, Sp^R^, Sm^R^ | This study |
| pBU267 | Suicide vector used to delete the *vtpB* homolog in OCN008; Cm^R^, Sp^R^, Sm^R^ | This study |
| pBU270 | Inducible expression plasmid used to express a wild type copy of *vasK* in OCN008; Cm^R^, Sp^R^, Sm^R^ | This study |
| pBU271 | Inducible expression plasmid used to express a wild type copy of *vtpR* in OCN008; Cm^R^, Sp^R^, Sm^R^ | This study |
