# Supplementary material for: Insight into the resilience and susceptibility of marine bacteria to T6SS attack by Vibrio cholerae and Vibrio coralliilyticus
Source: PLoS One. 2020 Jan 28;15(1):e0227864. doi: 10.1371/journal.pone.0227864 (PMC6986712; doi:10.1371/journal.pone.0227864)
Supplement: S2 Table — (DOCX) [file pone.0227864.s004.docx]

| Primer name | DNA sequence (5’ → 3’) | Source or citation |
| --- | --- | --- |
| pSW4426T-MCS-F | CTCAACGGGAATCCTGCTCTGCGAG | (Ushijima et al. 2016)^46^ |
| pSW4426T-MCS-R | ACTGCTTGGTGCCAGCCAATGAG | (Ushijima et al. 2016)^46^ |
| 008-vtpR-up-EcoRI-F | ATATATGAATTCACGAGAAGGCTTATCAAGCAGCGTA | This study |
| Vcor-vtpR-up-OEX-R | ACTTGTAGATGCAAAGCTTAGCTATAGAATCCATAGTTATATTTCCTTG | This study |
| Vcor-vtpR-down-OEX-F | GGATTCTATAGCTAAGCTTTGCATCTACAAGTAGATTAACCAGTGTCAT | This study |
| 008-vtpR-down-XbaI-R | ATGGTGAGAAAGCAGGTCTAGAAATTGATGAG | This study |
| 008vasK-up-EcoR1-F | ATATATGAATTCTCCTTCCGTACCGGGTGGTAT | This study |
| 008vasK-up-OEX-R | ATAGGTCGATGGTTTCTATAATATTTTTTAGCATAGTTATTCAGCCA | This study |
| 008vasK-down-OEX-F | GCTAAAAAATATTATAGAAACCATCGACCTATAAAAAAGTAAACTTA | This study |
| 008vasK-down-XbaI-R | ATATATTCTAGATCCACGCCGAGCAAACTACAAG | This study |
| vtpA-up-EcoRI-F | ATATATCGAATTCATTCGGCAATCATAAAGGCACAGGCTGT | This study |
| vtpA-up-OEX-R | AGTCTAATCTTAGTGTCATTTGACGTTGTTTCATTTTACTTTCCTGTT | This study |
| vtpA-down-OEX-F | GAAACAACGTCAAATGACACTAAGATTAGACTAATAAAAAAACACAAC | This study |
| vtpA-down-SpeI-R | ATATATACTAGTCTGGTTCTCTGCGGCCTTGCCATCTCTTTT | This study |
| vtpB-up-SpeI-F | ATATATACTAGTTACGTACCAAGTTTGGTTTAGGCCAGTTATT | This study |
| vtpB-up-OEX-R | AGTTGGCTTTGATGAAACGCTTGGCTATTTTCATTATGATTCTCCTT | This study |
| vtpB-down-OEX-F | GAAAATAGCCAAGCGTTTCATCAAAGCCAACTAATCCATTCTAA | This study |
| vtpB-down-SpeI-R | ATATATACTAGTTTGATGGTGCGATCTGGCTGGATACC | This study |
| vasK-XbaI-F | ATATATTCTAGAAGTCGTGGCTGAATAACTATGCTAAAAAATATTAT | This study |
| vasK-XbaI-R | ATATATTCTAGATTATAGGTCGATGGTTTCGGGTAGAGAGAAGTTAT | This study |
